# Development and Metabolomic Profiles of *Bactrocera dorsalis* (Diptera: Tephritidae) Larvae Exposed to Phytosanitary Irradiation Dose in Hypoxic Environment Using DI-SPME-GC/MS

**DOI:** 10.3390/insects15030177

**Published:** 2024-03-06

**Authors:** Changyao Shan, Baishu Li, Li Li, Qun Liu, Hang Zou, Tao Liu

**Affiliations:** Institute of Equipment Technology, Chinese Academy of Inspection and Quarantine, No. A3, Gaobeidianbeilu, Chaoyang District, Beijing 100123, China; 34424808@student.murdoch.edu.au (C.S.); libaishu@163.com (B.L.); lili@caiq.org.cn (L.L.); liuq@caiq.org.cn (Q.L.); zh1996nmg@126.com (H.Z.)

**Keywords:** *Bactrocera dorsalis*, modified atmosphere, irradiation, development, SPME technology, metabolites, radioprotective effects

## Abstract

**Simple Summary:**

The Oriental fruit fly, *Bactrocera dorsalis* (Hendel), significantly impacts agriculture in Southeast Asia and some Pacific islands as a major pest. Its widespread distribution, robust invasive ability, and detrimental effects on market access categorize it as a significant threat to numerous countries. Irradiation treatment is recognized as an effective and promising approach, considered as a potential alternative to the traditional phytosanitary treatment method, methyl bromide fumigation. However, the low-oxygen environment can influence the efficacy of irradiation treatment. The aim of this study is to investigate the impact of low-oxygen levels on the phytosanitary irradiation effects against larvae of *B. dorsalis*. The effects of normoxic (21% O_2_), hypoxic (5% O_2_), and super-hypoxic (0.5% O_2_) conditions on the development and metabolic profile of third-instar larvae of *B. dorsalis* were evaluated and compared at phytosanitary irradiation dose. Our research emphasizes the importance of lipid metabolism pathways and their associated metabolites in the irradiation tolerance of insects; moreover, neither hypoxic nor super-hypoxic conditions have increased the emergence rate of the evaluated fruit fly species under the current phytosanitary irradiation dose. These findings provide new insights into the mechanisms of radioprotection in insects under low-oxygen environments and advocate for international organizations and regulatory agencies to update guidelines on the application of phytosanitary irradiation under hypoxic conditions.

**Abstract:**

X-ray irradiation and modified atmospheres (MAs) provide eco-friendly, chemical-free methods for pest management. Although a low-oxygen atmospheric treatment improves the performance of some irradiated insects, its influence on the irradiation of quarantine insects and its impacts on pest control efficacy have yet to be investigated. Based on bioassay results, this study employed direct immersion solid-phase microextraction (DI-SPME) combined with gas chromatography-mass spectrometry (GC-MS) to determine metabolic profiles of late third-instar *B. dorsalis* larvae under normoxia (CON, Air), hypoxia (95% N_2_ + 5% O_2_, HY), super-hypoxia (99.5% N_2_ + 0.5% O_2_, Sup-HY), irradiation-alone (116 Gy, IR-alone), hypoxia + irradiation (HY + IR) and super-hypoxia + irradiation (Sup-HY + IR). Our findings reveal that, compared to the IR-alone group, the IR treatment under HY and Sup-HY (HY + IR and Sup-HY + IR) increases the larval pupation of *B. dorsalis*, and weakens the delaying effect of IR on the larval developmental stage. However, these 3 groups further hinder adult emergence under the phytosanitary IR dose of 116 Gy. Moreover, all IR-treated groups, including IR-alone, HY + IR, and Sup-HY + IR, lead to insect death as a coarctate larvae or pupae. Pathway analysis identified changed metabolic pathways across treatment groups. Specifically, changes in lipid metabolism-related pathways were observed: 3 in HY vs. CON, 2 in Sup-HY vs. CON, and 5 each in IR-alone vs. CON, HY + IR vs. CON, and Sup-HY + IR vs. CON. The treatments of IR-alone, HY + IR, and Sup-HY + IR induce comparable modifications in metabolic pathways. However, in the HY + IR, and Sup-HY + IR groups, the third-instar larvae of *B. dorsalis* demonstrate significantly fewer changes. Our research suggests that a low-oxygen environment (HY and Sup-HY) might enhance the radiation tolerance in *B. dorsalis* larvae by stabilizing lipid metabolism pathways at biologically feasible levels. Additionally, our findings indicate that the current phytosanitary IR dose contributes to the effective management of *B. dorsalis*, without being influenced by radioprotective effects. These results hold significant importance for understanding the biological effects of radiation on *B. dorsalis* and for developing IR-specific regulatory guidelines under MA environments.

## 1. Introduction

The oriental fruit fly, *Bactrocera dorsalis* (Hendel) (Diptera: Tephritidae), is a highly invasive species. It was first reported in East India in 1794 and has since widely been distributed throughout the Asia-Pacific region and much of sub-Saharan Africa. *B. dorsalis* is the polyphagous fruit fly with the broadest host range in the genus *Bactrocera*, which poses a severe threat to fruit and vegetable production [1,2]. This has led many nations and regions to list it as a quarantine pest. Both the adult and larval stages of *B. dorsalis* can inflict damage on fruits and vegetables, resulting in significant direct losses in agricultural yield and trade. The serious quarantine restrictions on potentially infested commodities can also lead to indirect economic losses [3]. Consequently, commodities susceptible to infestation must undergo phytosanitary treatments for disinfestation before export to regulated or quarantine areas.

Irradiation, including *γ*-rays, X-rays, and e-Beam, is an emerging phytosanitary treatment technique that has been effectively employed for the disinfection of fresh commodities, thereby fostering the growth of international agricultural trade [4]. Currently, it stands as the only method with a universally accepted dose to sterilize fruit flies on any host, as documented by the United States Department of Agriculture’s Animal and Plant Health Inspection Service (USDA-APHIS), with effective radiation doses ranging between 50 to 400 Gy [5,6]. Modified atmosphere packaging (MAP) is a well-proven technology for extending the shelf life of fresh commodities and preserving natural quality [7]. Therefore, the combination of MA/MAP with IR treatment is highly appealing to growers. However, this strategy poses a challenge for regulatory authorities because it is known to increase the radiotolerance of insects in a hypoxic environment [8]. *Follett* et al. have reported that the survival rate of the third-instar melon fly, *Bactrocera cucurbitae* (Cocquillett), subjected to IR treatment increases under MA conditions with oxygen levels of 1-3% [9]. Furthermore, it has been confirmed that various insects, including the cabbage looper, *Trichoplusia ni* (Hubner), the Caribbean fruit fly, *Anastrepha Suspensa* (Loew), and *B. dorsalis*, demonstrate an increased radiotolerance in hypoxic environments [10]. As a result, both the USDA-APHIS and International Plant Protection Convention (IPPC) regulations have imposed restrictions on the application of phytosanitary IR for fruits and vegetables under MAs.

Given the established fact that hypoxic environments enhance the radiotolerance of insects and other organisms, regulatory authorities’ concerns about using MA with phytosanitary IR without definitive evidence of its effectiveness in such conditions are understandable [11]. However, this does not automatically translate to the IR treatment being ineffective under hypoxic conditions. The primary objectives of phytosanitary IR are to prevent the development and reproduction, rather than to achieve the near-immediate mortality, of insects after treatment [12]. Viewed in this light, none of the studies mentioned above indicate the treatment’s failure under hypoxic conditions. Even if certain studies show a higher survival rate of insects within 24 h after IR in hypoxic conditions, the treatment is still considered successful if it prevents the emergence of adults or results in the sterility of the adult or F1 generation. Currently, the IPPC lacks specific quarantine guidelines for commodities in an MA subjected to phytosanitary IR. Therefore, until the efficacy of the currently recommended phytosanitary IR dose is verified under hypoxic conditions, it is wise not to adjust the IR dose in order to maintain the sensory quality of fresh commodities.

Furthermore, the mechanism of radioprotective effects on insects remains a “black box.” Generally, modern direct immersion microextraction sample preparation techniques have helped to make the analyte targets ready for sensitive treatment while subsequently revealing genuine information about molecular content [13,14]. Using the DI-SPME-GC-MS technique, this study analyzed the metabolome of third-instar *B. dorsalis* larvae exposed to IR in MA environments to gain molecular insights into the combined effects of HY or Sup-HY and IR. SPME is a recognized method for quick sample processing, especially in analytical biology, pharmaceuticals, and food research. When integrated with GC-MS, SPME significantly elevates the purity, consistency, and sensitivity of samples, surpassing conventional detection methodologies [15,16]. For instance, Liu et al., employed HS-SPME-GC-MS to analyze the metabolic changes in *B. dorsalis* larvae under different IR doses, and preliminarily provided eight potentially differential metabolites [17]. Alnajim et al. applied DI-SPME-GC-MS to extract and analyze hydrocarbons in the cuticle of both phosphine-resistant and sensitive strains of *Tribolium castaneum* (Herbst) and *Rhyzopertha dominica* (Fabricius), finding that the substantial presence of hydrocarbons could play a significant role in hindering the entry of phosphine into the bodies of resistant insects [18]. These studies suggest that SPME-GC-MS contributes to understanding potential radiobiological mechanisms and holds promise in the field of radiation detection.

## 2. Materials and Methods

### 2.1. Insect Rearing

The insects used in this study were collected from a mango orchard in the Guangxi Zhuang Autonomous Region, China, and reared in the laboratory of the Chinese Academy of Inspection and Quarantine (CAIQ). Third-instar larvae were transferred from mango fruits to a humid sterile sand for pupation, and the pupae were placed in cages (40 × 40 × 50 cm). Adult flies were fed a solid mixture of orange slices with sucrose and hydrolyzed yeast (3:1). Eggs were collected from adult females 2 weeks after emergence by allowing them to oviposit through mesh cage onto distilled water to maintain a temperature of 26 ± 1 °C. Eggs were collected in a 20-30 mL suspension containing eggs produced in 8–12 h. Larvae were reared on an artificial diet described by Vargas et al. (1984) under controlled conditions of 25 ± 2 °C, 70 ± 5% relative humidity, and a 12:12 (D:L) h photoperiod [19,20]. The emerged third-instar larvae were selected from the third to sixth generations and were utilized for all IR treatments.

### 2.2. Modified Atmosphere Treatments

Prior to IR treatment, MA treatments of *B. dorsalis* larvae were conducted using 3 L gas-tight air bags (Dalian Delin Gas Packaging Co., Ltd., Dalian, China). Each treatment involved collecting approximately 200 late third-instar larvae into a perforated plastic box. The larvae were then placed in a gas-tight bag, which was sealed through its opening. Subsequently, the exhaust valve was opened, and the bags were flushed with certified gas mixtures (95% N_2_ + 5% O_2_ and 99.5% N_2_ + 0.5% O_2_; provided by Beijing Green Oxygen Tiangang Technology Development Co., Ltd., Beijing, China) for 3 min. This procedure was repeated 3 times to purify the gas within the bags. Following this initial preparation, the gas-tight air bags containing the larvae were placed inside an incubator for approximately 12.4 h for the MA environments. For the HY + IR and Sup-HY + IR groups, gas-tight air bags containing the larvae and 1.5–1.6 L of MA were removed from the incubator after 12 h and then subjected to IR treatment. After the IR treatment, the gas-tight air bags for all groups were opened simultaneously to ensure consistent sealing time. In both the control group and the IR-alone treatment group, the larvae were placed in plastic boxes and enclosed in the gas-tight bags without requiring any additional gas flushing procedures.

### 2.3. Irradiation Treatments

X-ray IR treatment was conducted in the CAIQ Irradiation Processing Laboratory using the RS-2000 ProX irradiator (Rad Source-Technologies, Inc., Coral Springs, FL, USA) at room temperature. Operating parameters were established at 220 kV and 17.6 mA. Larvae, enclosed in a gas-tight air bag, were positioned in an exposure chamber with dimensions of 17 inches in width, 15 inches in depth, and 17 inches in height. A RadCal dosimeter (model 2086, RadCal Corp., Monrovia, CA, USA) equipped with a 10 × 6^−0.6^ ion chamber was positioned near the test insects. Following each irradiation treatment, the dosimeter recorded the cumulative dose. The irradiator stopped operation when the cumulative dose reached the minimum dose amount (116 Gy) [17,21]. The dose rate observed in this study was approximately 5.0 Gy/min, with the IR duration lasting about 23.2 min.

### 2.4. Develpomental Test

**Figure 1:**
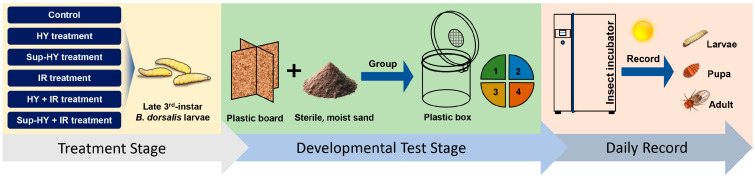
Schematic diagram of the technical process for the development test of the late third-instar *B. dorsalis* (Diptera: Tephritidae) larvae under different treatment conditions (Control, HY, Sup-HY, IR, HY + IR, and Sup-HY + IR).

Following the treatments, 160 late third-instar *B. dorsalis* larvae were randomly sampled from each group, including the control and all treatment groups. Each larva was individually placed in a plastic box (5 cm diameter, 3 cm height) with sterile moist sand inside an environmental control chamber (KBF 720; WTC Binder, Tuttlingen, Germany). Rearing conditions were set at 25 °C, 60% RH, and a 12:12 h (L:D) photoperiod. The developmental progress of each individual was observed and recorded daily at 9:00 a.m. over a 2-week period. Figure 1 illustrates the processes adopted to achieve the aforementioned objectives [22].

The age-stage-specific proportions represent the probability of third-instar *B. dorsalis* larvae growing to a specific stage and were calculated as follows.

$$Proportion=N_{xj}/N$$

where *N* is the number of individuals used at the beginning of the developmental study, and *N_xj_* is the number of individuals growing to day *x* and stage *j* [23].

### 2.5. Sample Preparation and Extraction Using DI-SPME

After completing all treatment procedures, 3 late third-instar *B. dorsalis* larvae were immediately selected from each of the 6 experimental groups (CON, IR-alone, HY, Sup-HY, HY + IR, and Sup-HY + IR). These larvae were moved into a 2 mL microtube (Eppendorf, Germany) and promptly frozen using liquid nitrogen. They were stored in an ultra-low-temperature freezer (MDF-U73V, Sanyo Electric Co. Ltd., Osaka, Japan) at −80 °C for the subsequent experiments. These samples were intended for studying the impact of different treatment methods on the metabolites of *B. dorsalis* larvae. The frozen samples of the insects were ground into powder within the microtube and immediately suspended in 1.3 mL of HPLC-grade acetonitrile (≥99.9%, Sinopharm Chemical Reagent Co., Ltd., Shanghai, China). The microtubes were centrifuged at 4000 rpm for 3 min at 4 °C using a centrifuge (5417R, Eppendorf, Hamburg, Germany). The supernatant (1.3 mL) was transferred to an amber-colored chromatography vial with PTFE-coated septa (Supelco, Darmstadt, Germany). The SPME fiber, coated with 50/30 µm of Carboxen/DVB/PDMS (Sigma-Aldrich, Bellefonte, PA, USA), was introduced into the samples for extraction and conditioned at 25 ± 1 °C for 1 h. Subsequently, a GC-MS analysis was conducted on the DI-SPME, with a desorption time of 15 min. The samples were analyzed in triplicate for biological replicates.

### 2.6. Metabolites Analysis with GC-MS

DI-SPME samples were analyzed using an Agilent 8890 gas chromatograph (GC) coupled with an Agilent 5977B mass spectrometry detector (MSD) system in splitless mode. The HP-5MS capillary column (30 m × 0.25 mm, 0.25 μm; Agilent J&W Scientific, Santa Clara, CA, USA) was used for separation. Helium gas (>99.999%) was employed as the carrier gas at a constant flow rate of 1.0 mL/min. The GC-MS injection temperature was maintained at 270 °C. The initial temperature was set at 60 °C and held for 2 min, then ramped at 7 °C/min to 200 °C, 5 °C/min to 300 °C, and was finally increased to 320 °C at a rate of 50 °C/min, and held for 3 min. The ion source temperature was set at 230 °C, and the MSD transfer line temperature was set at 280 °C. Electron impact ionization (70 eV) was utilized in full-scan mode (*m*/*z* 30-550), with the default settings for the other parameters. The solvent delay time was set to 5 min, and the total run time was 50.4 min. Additionally, quality control samples were first analyzed during the experimental process to assess stability.

### 2.7. Statistical Analysis

GC-MS data were first processed with Agilent MassHunter Qualitative Analysis Software (Version 10.0), with peak identification augmented by Kovat’s retention index (RI) method [24]. The generated three-dimensional dataset comprised the sample details, retention time (RT), and peak intensities. Univariate and multivariate analyses, as well as a pathway analysis, were performed using the R platform (Version 4.1.3) with the prcomp, MixOmics, and clusterProfiler packages. These analyses included a one-way ANOVA, principal component analysis (PCA), partial least squares discriminant analysis (PLS-DA), heatmap analysis, and KEGG enrichment analysis. Additionally, unless otherwise specified, a significance level of 0.05 was employed for assessing differences.

## 3. Results

### 3.1. Develpment of Bactrocera dorsalis (Diptera: Tephritidae) Larvae in a Hypoxic Environment with Phytosanitary Irradiation

**Figure 2:**
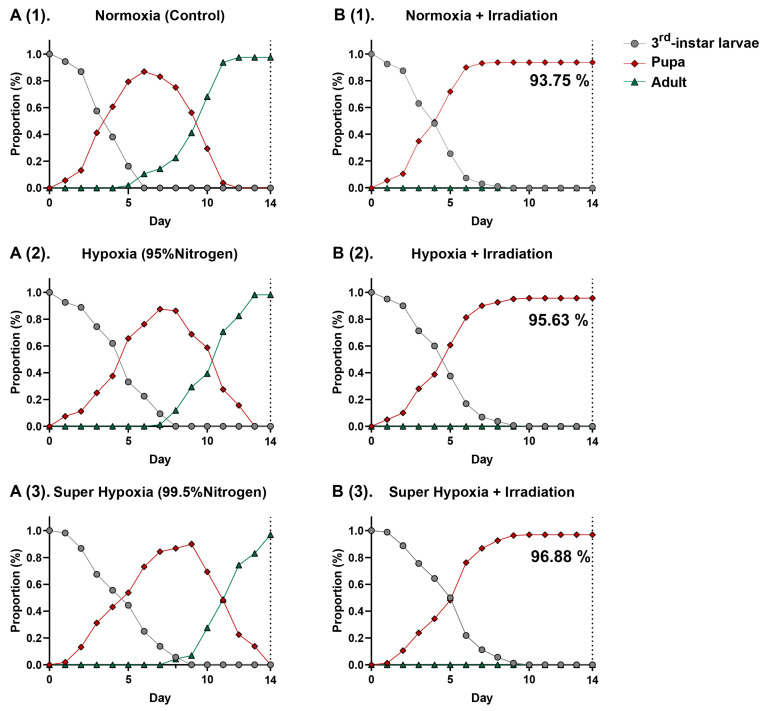
Proportion of *B. dorsalis* (Diptera: Tephritidae) larvae in the control (**A(1)**), hypoxia (**A(2)**), super hypoxia (**A(3)**), irradiation (**B(1)**), hypoxia + irradiation (**B(2)**) and super-hypoxia + irradiation (**B(3)**) treatment groups.

As shown in Figure 2(A(1)–A(3)),(B(1)–B(3)), distinct differences are observed in the curves of *B. dorsalis* third-instar larvae across the six different conditions. Within these curves, overlaps are observed, suggesting variations in the developmental rates among individuals. MA environments, including HY and Sup-HY, markedly delay the development of *B. dorsalis* larvae, extending their pupation phases (Table 1). However, the developmental postponement effects of Sup-HY and HY on the *B. dorsalis* third-instar larvae are nearly identical, possibly due to the reduced sensitivity of the *B. dorsalis* to oxygen when the concentration drops to a certain level. Under normoxic, hypoxic, and super-hypoxic conditions, IR effectively inhibits emergence. The results indicate that the current phytosanitary irradiation dose (116 Gy) fully meets the standard for *B. dorsalis* quarantine treatment. These three approaches sustain the *B. dorsalis* larvae pupation rate near 95%. Notably, the IR treatment under MA environments had slight antagonistic effects. This might be attributed to the reduced sensitivity of the larvae to IR under HY or Sup-HY, resulting in an increased pupation rate in the HY/Sup-HY + IR treatment group compared to the normoxic IR treatment group, and significantly mitigating the delaying effect of IR on the larval developmental periods, as shown in Table 1. This novel finding, not previously reported, is hypothesized to be related to metabolic changes within the larvae induced by the hypoxic environment and a potential radioprotective effect.

### 3.2. Metabolic Profiles Analyzed via GC-MS

As illustrated in Table 2, we identified a total of 25 metabolites from 6 different groups—CON, IR-alone, HY, Sup-HY, HY + IR, and Sup-HY + IR—based on a compound matching degree of over 80% and RI. Specifically, 23 metabolites were identified in the CON group, 12 in the IR-alone group, 19 in the HY group, 16 in the Sup-HY group, 13 in the HY + IR group, and 10 in the Sup-HY + IR group. While some metabolites were ubiquitously expressed across all groups, others exhibited significant variations in specific groups. A one-way ANOVA, followed by Fisher‘s LSD post hoc analysis, revealed significant differences (*p* < 0.05) in the GC-MS response of 24 compounds, as depicted in Figure S1. Metabolites such as 4-methyl-heptadecane, 1-docosene, 2-pentadecanone, nonadecane, and squalene were found to be upregulated in the IR, HY, Sup-HY, HY+IR, and Sup-HY + IR groups. Conversely, metabolites like n-hexadecanoic acid, sitosterol, (E)-2-decenal, 2-dodecenal, and tetradecanal manifested a downregulation following these treatments. Notably, the majority of metabolites that were downregulated were co-clustered in the cluster analysis, suggesting a potential similarity in functions within the metabolic pathways of the *B. dorsalis* larvae.

**Figure 3:**
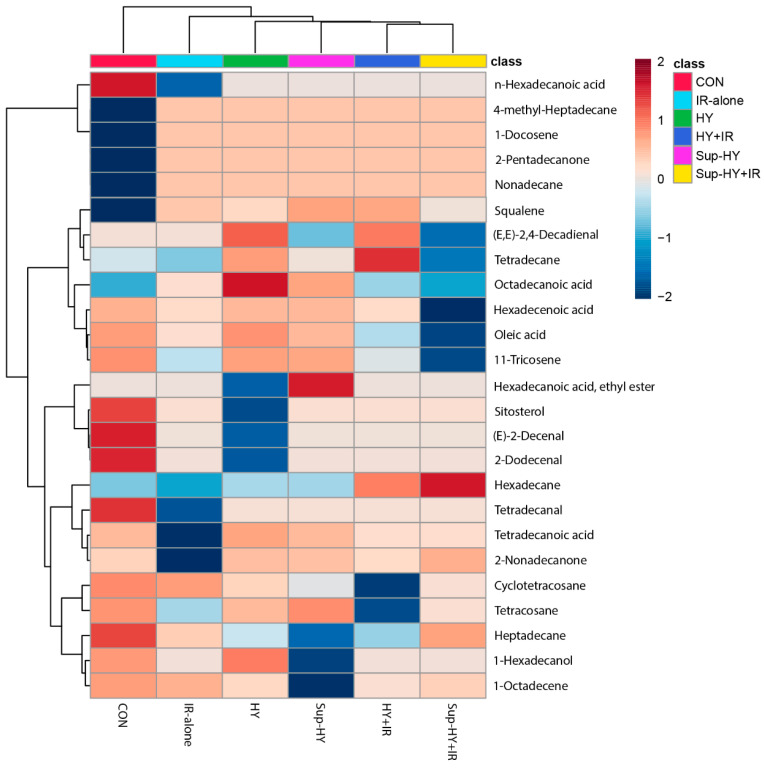
Heatmap displaying the abundance changes of the metabolites significantly influenced by different treatment methods, with significance determined via one-way ANOVA at *p* < 0.05.

To visually represent the expression patterns of the metabolites across different groups, a clustering heatmap was constructed to cluster the identified metabolites based on the similarity of their content changes. In this heatmap, each cell represents the average content of a metabolite, with blue indicating downregulation, and red indicating upregulation. As shown in Figure 3, the 24 metabolites subjected to hierarchical clustering in each group (CON, IR, HY, Sup-HY, HY + IR, and Sup-HY + IR) showed different regulatory directions significantly. This provides a clear representation, revealing the variations in the metabolite patterns of the *B. dorsalis* larvae under different treatment conditions. To gain further insights into the metabolic responses of *B. dorsalis* larvae to these five treatment methods, we will further employ multivariate statistical methods for analysis.

### 3.3. Multivariate Statistical Analysis of B. dorsalis Larvae Metabolites

**Figure 4:**
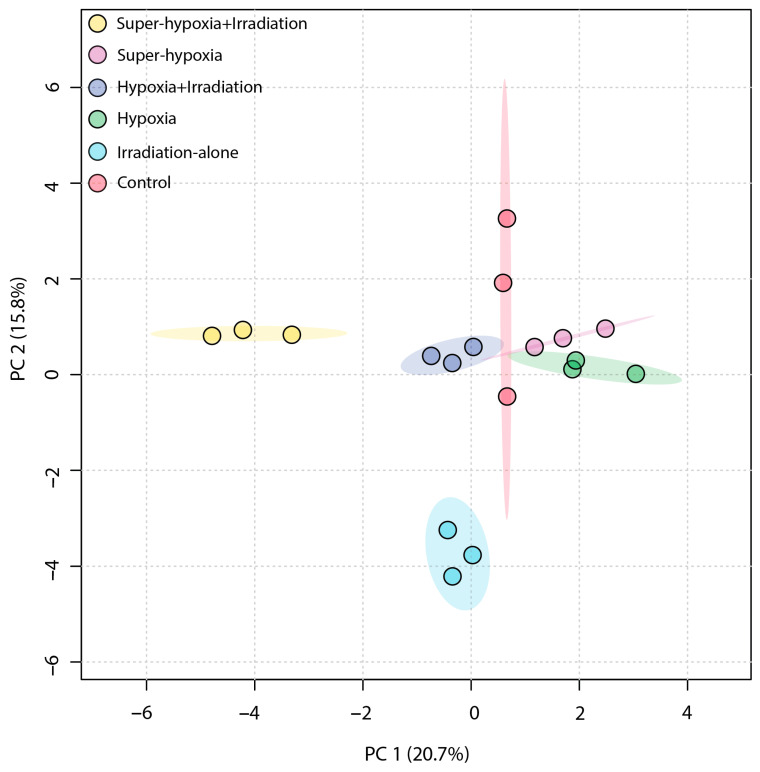
PCA score plot of the metabolic profiles in the *B. dorsalis* (Diptera: Tephritidae) larvae exposed to different MAs under IR (116 Gy).

**Figure 5:**
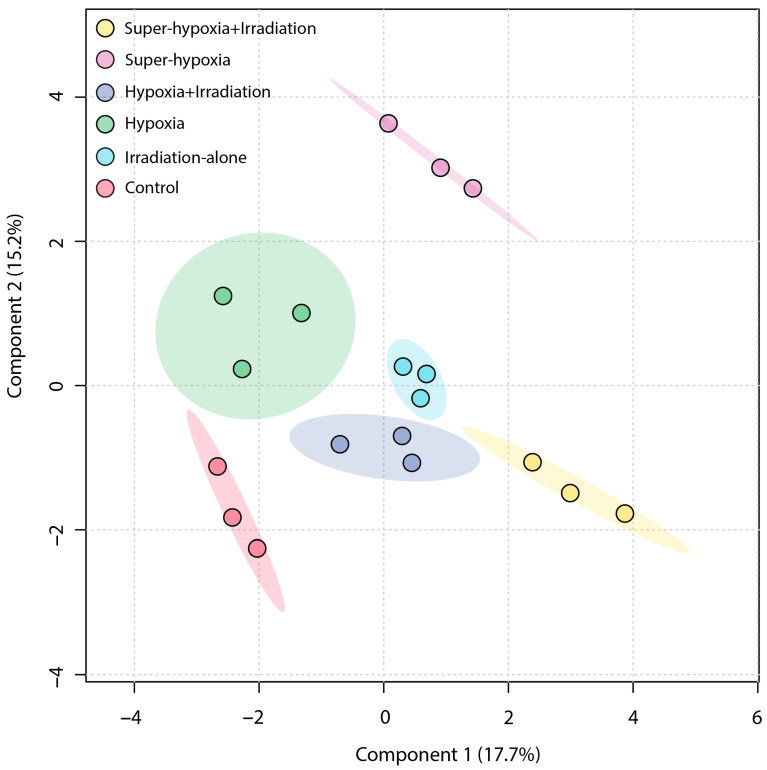
PLS-DA score plot of the metabolic profiles in the *B. dorsalis* (Diptera: Tephritidae) larvae exposed to different MAs under IR (116 Gy).

An unsupervised PCA was employed to obtain a general overview of the clustering information between groups, with the grouping in a PCA score plot being based on the similarities between the metabolic profiles of the samples. As shown in Figure 4, the first principal component accounted for 20.7% of the total variance in the original data, while the second principal component explained 15.8%. On the first principal component, a distinct separation was observed between the IR-alone group and the Sup-HY + IR group. However, the 95% Hotelling’s T-squared ellipse overlapped between the CON and the HY, Sup-HY, and HY + IR groups. In comparison to the PCA, the partial least squares discriminant analysis (PLS-DA) model, being a supervised learning method, demonstrated superior discriminatory power. The score plot (Figure 5) shows that the IR-alone, HY, Sup-HY, HY + IR, and Sup-HY + IR groups were clearly separated from the control group. Meanwhile, clear separations were observed among the IR-alone, HY, Sup-HY, HY + IR, and Sup-HY + IR groups in the PLS-DA model. These findings indicate that all five treatments—IR-alone, HY, Sup-HY, HY + IR, and Sup-HY + IR—significantly altered the metabolic profiles of the *B. dorsalis* larvae. Consequently, we can employ statistical methodologies to further identify the key differential metabolites between the treatment groups.

### 3.4. Statistical Analysis and Differentially Regulated Metabolites

“Differential metabolites” were defined as substances that were identified among different samples but showed significant variations in their respective concentrations. For differentials among the CON vs. IR-alone, CON vs. HY, CON vs. Sup-HY, CON vs. HY + IR, and CON vs. Sup-HY + IR, separate PLS-DA analyses were conducted on their respective metabolic datasets. Metabolites that contributed to the differentiation were identified based on a variable importance in the projection (VIP) value of the PLS-DA model (threshold > 1).

**Figure 6:**
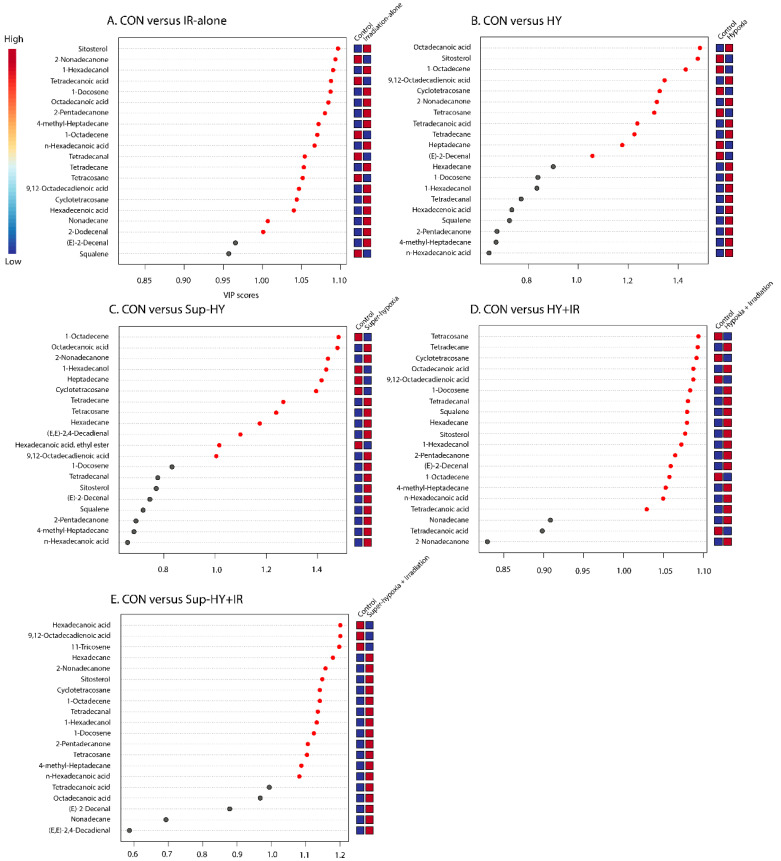
A variable importance plot showing the contribution of each metabolite to the first component (ranked by VIP scores) in the CON vs. IR (**A**), CON vs. HY (**B**), CON vs. Super-HY (**C**), CON vs. HY + IR (**D**), and CON vs. Sup-HY + IR (**E**) groups.

Furthermore, metabolites specific to the IR-alone, HY, Sup-HY, HY + IR, and Sup-HY + IR groups were individually identified, as depicted in Figure 6A–E. By consolidating the results from the Student’s *t*-test (with a threshold of *p* < 0.05) and a VIP value greater than 1.0, we identified 18, 9, 10, 17, and 15 key differential metabolites in the *B. dorsalis* larvae treated with IR-alone, HY, Sup-HY, HY + IR, and Sup-HY + IR, respectively.

**Figure 7:**
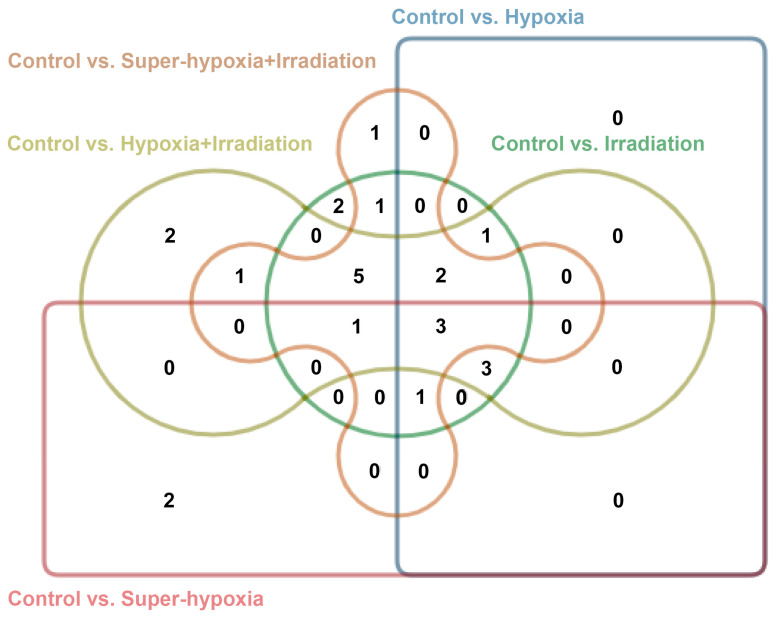
Edward’s Venn diagram of the CON vs. IR, CON vs. HY, CON vs. Sup-HY, CON vs. HY + IR, and CON vs. Sup-HY + IR groups. Different colors represent 5 groups of data sets, and numbers indicate the quantity of elements in the overlapping areas.

Importantly, 2-dodecenal and nonadecane were unique to the IR-alone group; hexadecanoic acid, ethyl ester, and heptadecane were specific to the Sup-HY group; (E)-2-decenal and squalene were exclusive to the HY + IR group; and 11-tricosene was only detected in the Sup-HY + IR group, as illustrated in Figure 7.

### 3.5. Key Metabolic Pathway Analysis

As shown in Table 3, compared to the CON, the IR-alone, HY, Sup-HY, HY + IR, and Sup-HY + IR groups identified five, three, two, five, and five differential metabolic pathways, respectively. This suggests that the metabolic response of *B. dorsalis* larvae to the IR treatment is more complex than that to the MA treatment. Among these six groups, two common metabolic pathways were observed: the biosynthesis of unsaturated fatty acids and fatty acid biosynthesis.

**Figure 8:**
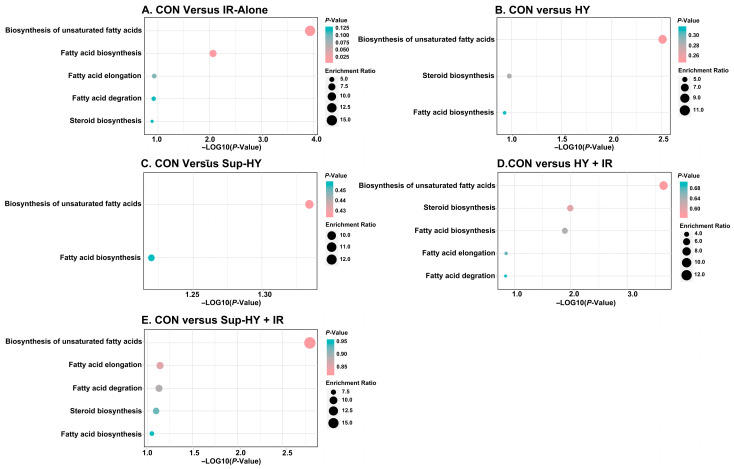
Metabolome view map of the significant metabolic pathways characterized in the *B. dorsalis* (Diptera: Tephritidae) larvae exposed to different modified atmospheres under a phytosanitary irradiation dose. (**A**) CON vs. IR-alone, (**B**) CON vs. HY, (**C**) CON vs. Sup-HY, (**D**) CON vs. HY + IR, and (**E**) CON vs. Sup-HY + IR.

Based on the criteria of *p* (>0.05) and impact value (>0.1), the key metabolic pathways for the CON vs. IR-alone, CON vs. HY, CON vs. Sup-HY, CON vs. HY + IR, and CON vs. Sup-HY + IR groups were identified as five, two, zero, five, and zero, respectively, as depicted in Figure 8. Specifically, in the CON vs. IR-alone group, the pathways of the biosynthesis of unsaturated fatty acids, fatty acid biosynthesis, fatty acid elongation, fatty acid degradation, and steroid biosynthesis were deemed critical, with corresponding impact values of 0.117, 0.153, 0.124, 0.127, and 0.137. In the CON vs. HY group, fatty acid biosynthesis and steroid biosynthesis emerged as the key pathways, with impact values of 0.109 and 0.122, respectively. For the HY + IR group, the key metabolic pathways included the biosynthesis of unsaturated fatty acids, steroid biosynthesis, fatty acid biosynthesis, fatty acid elongation, and fatty acid degradation, with impact values of 0.141, 0.164, 0.184, 0.148, and 0.152, respectively.

Although the differential metabolites in the Sup-HY and Sup-HY + IR groups were enriched in two and five metabolic pathways, respectively, their impact on these pathways was not significant. Notably, the pathways of fatty acid elongation and fatty acid degradation emerged as characteristic metabolic pathways in the *B. dorsalis* larvae following IR treatment (IR-alone, HY + IR, and Sup-HY + IR). Furthermore, IR treatments under both the hypoxic and super-hypoxic conditions influenced five metabolic pathways, the biosynthesis of unsaturated fatty acids, fatty acid biosynthesis, fatty acid elongation, fatty acid degradation, and steroid biosynthesis. The findings seem to correlate with the developmental results of the six groups of *B. dorsalis* larvae described in Section 3.1. Hence, these five metabolic pathways might play an important role in influencing the sensitivity of *B. dorsalis* larvae to IR.

### 3.6. Comparison of the Main Differential Metabolic Responses

Through a detailed analysis of the affected metabolites and their associated pathways, we aim to understand the differential metabolic responses of *B. dorsalis* larvae under various treatments, including IR-alone, HY, Sup-HY, HY + IR, and Sup-HY + IR.

**Figure 9:**
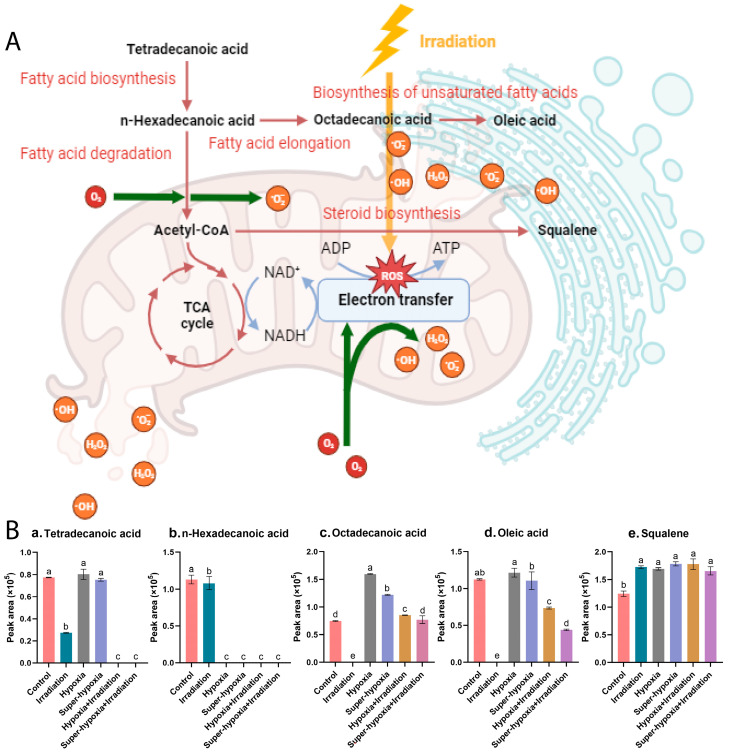
(**A**) The integrated schematic diagram of the key metabolic pathways and metabolites in the *B. dorsalis* (Diptera: Tephritidae) larvae exposed to different modified atmospheres under a phytosanitary irradiation dose, and (**B**) the changes in peak areas of 5 key compounds ((**a**). tetradecanoic acid, (**b**). n-hexadecanoic acid, (**c**). octadecanoic acid, (**d**). oleic acid and (**e**). squalene) across different treatment conditions.

Based on the KEGG database, we constructed a metabolic pathway network, highlighting the interconnections among the aforementioned five core metabolic pathways and their key compounds, as illustrated in Figure 9A,B. These pathways reveal the primary metabolic variations and shared responses of *B. dorsalis* larvae under diverse treatment conditions. Notably, the levels of tetradecanoic acid, n-hexadecanoic acid, octadecanoic acid, and oleic acid were significantly reduced in all IR-treated groups. These fatty acids are essential components of several metabolic pathways, including the biosynthesis of unsaturated fatty acids, fatty acid biosynthesis, fatty acid elongation, and fatty acid degradation. This significant decrease can be attributed to the surge in ROS production induced by IR treatment, resulting in localized mitochondrial autophagy [25]. Consequently, mitochondrial energy metabolism is inhibited, prompting the swift oxidation of fatty acids into acetyl-CoA that subsequently enters the TCA cycle for energy generation.

In both hypoxic treatment groups, HY and Sup-HY, there was a notable decrease in n-hexadecanoic acid levels and a corresponding increase in octadecanoic acid levels. This pattern suggests the activation of both the fatty acid degradation and fatty acid elongation metabolic pathways. The presence of oleic acid suggested the activation of the biosynthesis of unsaturated fatty acids pathway. However, there was no significant difference in oleic acid concentration compared to the control group. The likely cause of this observation is the activation of Hypoxia-Inducible Factor-1 (HIF-1) in both the hypoxic and super-hypoxic conditions. The activation of HIF-1 boosts the expression of antioxidative enzymes, thereby regulating ROS levels within the organism [26]. Consequently, oleic acid experiences reduced oxidative stress, diminishing the formation of lipid peroxides.

Regarding the IR treatment under the HY and Sup-HY conditions, certain fatty acid levels decreased compared to other groups, presumably because of the combined effects of low-oxygen and IR. The notable reduction in tetradecanoic acid and n-hexadecanoic acid levels indicates a gradual shift in the *B. dorsalis* larvae’s primary energy metabolism from carbohydrates to lipids for efficient ATP production. This metabolic shift typically indicates that the organism is exploiting its “energy reserves” to achieve a temporary stress-adaptive capacity under adverse conditions. Consequently, we hypothesize that the fatty acid metabolism might be a crucial pathway for the *B. dorsalis* larvae to initiate their antioxidative defense mechanism, assisting in the clearance of ROSs induced by IR and reducing their IR sensitivity. Notably, this antioxidative effect appears to amplify as the oxygen concentration decreases, as evidenced by the results in Section 3.1. This observation offers a persuasive explanation for the developmental experiments of the *B. dorsalis* in our study. While the precise mechanism behind such radioprotective effects remains a ‘black-box’, our findings could offer a valuable reference for subsequent research.

The observed alteration in the squalene concentration is noteworthy, given its apparent sensitivity to both low-oxygen and IR conditions. In biological systems, squalene is recognized for augmenting the activity of superoxide dismutase (SOD) [27]. Consequently, elevated squalene levels might correlate with the impact of hypoxia and irradiation on ROS concentrations in *B. dorsalis* larvae.

## 4. Discussion

Until recently, there have been only a few studies on the impacts of IR in hypoxic conditions on the reproduction and development of *B. dorsalis* [8,10,28]. The hypoxic environment diminishes the efficacy of IR on certain insects, leading the IPPC to withhold support for IR for the quarantine treatment of MAP commodities [12]. This study, however, demonstrates that the current phytosanitary IR dose is effective in inhibiting *B. dorsalis* larvae even under HY/Sup-HY conditions (Figure 2), lending support to the administrative adjustments by the USDA-APHIS revising the minimum O_2_ level for treatments from 18% to 10% [29]. Our research findings also provide a robust basis for the IPPC to reassess the restrictions on phytosanitary IR applications for *B. dorsalis* under hypoxic conditions.

IR-induced DNA damage, a significant factor causing male sterility and impacting insect growth [30,31], might suggest the activation of DNA repair genes under hypoxic conditions. However, the RNA-seq analysis conducted by Wang et al. on DNA repair-related genes in the cowpea weevil (*Callosobruchus maculatus*), including XRCC1, XRCC3, RAD23, and RAD51, indicates otherwise [8]. Their findings challenge the hypothesis that hypoxia aids in DNA-damage repair, suggesting that the mechanisms behind radioprotective effects in hypoxic conditions warrant further investigation. Our study first employed the DI-SPME-GC-MS technique, a validated and effective tool for metabolomic analysis, to explore this area.

Topological analysis showed that treatments like IR-alone, HY, Sup-HY, HY + IR, and Sup-HY + IR primarily exhibit changes in the lipid metabolism pathways compared to the control group, especially in unsaturated fatty acids and fatty acid biosynthesis. These pathways are crucial for maintaining biological tissue structure and function [32,33]. Interestingly, there was an overlap in the differential metabolic pathways observed across these five treatment methods, suggesting a certain correlation in the mechanisms of action of IR-alone, HY, Sup-HY, HY + IR and Sup-HY + IR on *B. dorsalis* larvae. This also implies that the combined impacts of IR and MAs extend beyond a mere additive “1 + 1” effect.

In this study, significant changes were observed in the free fatty acids of *B. dorsalis* larvae across different treatment groups. These fatty acids are not only essential for maintaining cell membrane fluidity and permeability but are also vital in anti-oxidative stress [34,35,36]. Notably, n-hexadecanoic acid was consistently downregulated across all treatment groups, indicating a marked inhibition of the fatty acid biosynthesis pathway. As depicted in Figure 9B, n-hexadecanoic acid, a key differential regulatory metabolite, holds a central position in the differential metabolic pathways of the *B. dorsalis* larvae subjected to IR-alone, HY, Sup-HY, HY + IR, and Sup-HY + IR treatments. N-hexadecanoic acid, a precursor to many long-chain and unsaturated fatty acids, can be transformed into significant amounts of free fatty acids like octadecanoic acid through the fatty acid elongation pathway, and their accumulation in the endoplasmic reticulum may inhibit certain protein synthesis pathways, negatively affecting insect growth and development [37,38]. Furthermore, squalene is synthesized in the endoplasmic reticulum through the steroid biosynthesis pathway and promotes biological oxidation and metabolism, thereby enhancing the defense and stress-response capabilities of insects [39]. Currently, the specific changes in the lipid metabolism within the insects following the IR-alone, HY, Sup-HY, HY + IR and Sup-HY treatments are not fully understood. Therefore, further studies are needed to comprehensively understand the roles of lipid metabolic pathways.

Research suggests that diminished metabolic activity can counteract the oxidative stress caused by IR under hypoxic conditions. As previous studies have highlighted, both IR and MA treatments impact the mitochondrial electron transport chain and the TCA cycle [40,41,42]. Thus, carbohydrates might have a limited role in the insect energy metabolism post IR and MA treatments. This finding further strengthens the conclusion that lipid metabolic pathways have a significant impact on insects after treatments such as IR-alone, HY, Sup-HY, IR + HY, and IR + Sup-HY. Furthermore, Pablo et al.’s research showed that the development of the Mediterranean fruit fly (*Ceratitis capitata*) is regulated by changes in oxygen consumption rates and the levels of primary energy molecules, such as lipids, glycogen, and trehalose. Yan et al. analyzed the expression patterns and functions of fatty acid synthase genes (*SlFAS1* and *SlFAS2*) in the larvae of the tobacco cutworm (*Spodotera litura*), demonstrating the crucial role of fatty acid synthesis and storage in insects’ metabolism and metamorphic development [43,44]. These findings support the results observed in this study of the developmental delays in *B. dorsalis* larvae under hypoxic conditions, as depicted in Figure 2(A(1)–A(3)); the decreased pupation rate and hindered emergence, as shown in Figure 2(B(1)–B(3)), suggest that IR further impairs metabolic activity in insects, thereby hindering their normal development within the expected timeframe.

Based on metabolomics, this study provides a preliminary mechanistic explanation for the enhanced tolerance of *B. dorsalis* larvae to IR under hypoxic stress for the first time. It is well established that the metabolic rate of *B. dorsalis* larvae significantly decreases in hypoxic conditions [8,45]. However, under hypoxic conditions, while both the carbohydrate and lipid metabolisms are limited, cells favor the fatty acid metabolism—a more ATP-productive pathway—with the primary source of these fatty acids being the oxidation of intracellular lipid droplets [46,47,48]. In the early phases of hypoxic stress, cells quickly amass substantial ROSs, mainly from an overload of electrons in the electron transport chain due to a lack of oxygen [49,50]. Furthermore, IR not only damages DNA structures but also disrupts the electron transport chain, resulting in substantial ROS production [8,30,51]. Excessive ROSs can act as signaling molecules to enhance the activity of antioxidative enzymes, maintaining an ROS balance in organisms [52]. Key factors in the oxygen-sensing pathway, such as HIF-1 and nuclear factor erythroid-derived 2-like 2 (Nrf2), induced and upregulated by hypoxic stress, regulate the gene expression of antioxidative enzymes. This enhances the organism’s oxidative resistance, improving stress resistance during development. Specifically, HIF-1 activation inhibits the TCA cycle, increases the production of reducing molecules in organisms, and promotes lipid degradation [53]. Meanwhile, Nrf2 translocates to the cell nucleus, binding to the antioxidant response element (ARE), thereby activating the expression of antioxidative and detoxifying genes [54]. Moreover, studies have shown that hypoxic stress also can induce mitochondrial autophagy mediated by BNIP3 and FUNDC1, further alleviating oxidative damage caused by ROSs [55,56].

This study has potential limitations. We evaluated the effects of IR based on observations of naked *B. dorsalis* larvae, which differs from typical phytosanitary treatments on whole fruits. However, our simplified experimental design ensured each larva received a uniform and precise IR dose, allowing us to directly link metabolic changes to the irradiation treatment. Our evaluation may be conservative, and underestimate the impact of fruits on these treatments, as (1) fruits provide a complex treatment environment for insects, influenced by both biotic and abiotic factors. This increases the risk related to the fruit’s impact on metabolic changes in insects [57]; (2) Fruit tissues and structures attenuate and scatter X-rays, affecting the dose of IR subjected to insects [58,59]; (3) The efficacy of IR treatment may be influenced by the behavior of insect larvae. For example, some third-instar larvae are often found inside fruit galleries, while others, especially those nearing pupariation, seek shelter outside the fruit [60]. This study did not employ typical phytosanitary treatment programs involving whole fruits with insects, as our focus was more on the metabolic changes in insects resulting from different treatment methods. The execution of experiments requires strong stability and reproducibility; however, introducing fruits could lead to numerous extraneous variables, thereby interfering with the accurate assessment of experimental results.

In conclusion, we utilized the DI-SPME-GC-MS technique to first explore the mechanism by which hypoxic stress enhances the radiotolerance of *B. dorsalis* larvae from a lipid metabolism perspective. This research contributed to a deeper understanding of the radioprotection mechanisms in insects. Furthermore, our findings indicate that the *B. dorsalis* larvae, as a non-model insect, exhibit a conservative response pathway when subjected to an MA or IR stress. In addition, insects, as complex biological systems, have stress responses to external environmental threats that are co-regulated by multiple metabolic pathways, including those of amino acids, sugars, and lipids. Therefore, relying solely on a single metabolic pathway can hardly provide a comprehensive interpretation of these responses. Future research should focus more on the interactions between different metabolic pathways to further elucidate the radioprotection mechanisms in insects.

## 5. Conclusions

X-ray irradiation has a lethal effect on many insects. However, a hypoxic environment can weaken the impact of IR treatment, reducing the sensitivity of *B. dorsalis* larvae to IR and subsequently disrupting their normal development and metabolism. Notably, IR can induce significant changes in the lipid metabolism pathways of *B. dorsalis* larvae, as well as in the associated levels of free fatty acids, which are moderated in hypoxic conditions. Future research should emphasize the role of lipid metabolism pathways and their associated metabolites in evaluating the IR tolerance of insects, providing support for developing new post-harvest pest management strategies.

Furthermore, our preliminary research reveals the mechanisms by which the larvae of the *B. dorsalis* enhance their tolerance to IR under hypoxic conditions. These insights have practical implications for quarantine treatments. By limiting the oxygen content in the environment, the efficacy of IR treatments can be optimized, providing an important theoretical and data basis for the combined application of an MA and IR in quarantine procedures. Our findings indicate that either HY or Sup-HY environments influence the pupation rate and developmental period of the evaluated fruit fly species at the phytosanitary IR dose. Nevertheless, none of the *B. dorsalis* larvae treated under these conditions developed into adults. As a result, this study advocates for the updating of guidelines by international organizations and regulatory agencies regarding phytosanitary IR applications for *B. dorsalis* in low-oxygen environments.

## Figures and Tables

**Table 1:** Developmental periods (days) (mean ± SE) of the *B. dorsalis* (Diptera: Tephritidae) larvae in the different treatment groups.

Stage	Conditions	Control	Irradiation
Larvae	Normoxia	3.09 ± 1.25 bA	3.55 ± 1.52 bB
	Hypoxia (5%)	4.00 ± 1.51 aA	4.02 ± 1.74 abA
	Sup-Hypoxia (0.50%)	4.05 ± 2.07 aA	4.23 ± 1.94 aA
Pupa	Normoxia	5.48 ± 0.70 c	N.A.
	Hypoxia (5%)	5.89 ± 0.51 b	N.A.
	Sup-Hypoxia (0.50%)	6.52 ± 0.78 a	N.A.
Adult	Normoxia	N.A.	N
	Hypoxia (5%)	N.A.	N
	Sup-Hypoxia (0.50%)	N.A.	N

Different lowercase letters (a, b and c) indicate significantly different means within a row (*p* < 0.05); and different uppercase letters (A and B) in the same columns are significantly different at the 0.05 level based on the one-way ANOVA and Tukey’s honestly significant difference (HSD) test. N.A. = Not Applicable; N = No data.

**Table 2:** Metabolite profile of *B. dorsalis* (Diptera: Tephritidae) larvae exposed to MAs under IR (116 Gy).

^1^ RT	^2^ RI	Metabolites	Peak Area ± ^4^ SD						*p*-Value	CAS
			Normoxia		Hypoxia		Hypoxia + Irradiation (116 Gy)			
			Control	Irradiation-Alone	95% N_2_	99.5% N_2_	95% N_2_	99.5% N_2_		
12.130	1141.22	(E)-2-Decenal	0.255 ± 0.013 a	^3^ N.D. c	0.212 ± 0.010 b	N.D. c	N.D. c	N.D. c	1.75 × 10^−13^	003913-81-3
13.206	1256.58	(E,E)-2,4-Decadienal	N.D. c	N.D. c	0.241 ± 0.041 a	0.221 ± 0.020 ab	0.240 ± 0.026 a	0.237 ± 0.014 b	7.27 × 10^−14^	025152-84-5
14.093	1322.34	2-Dodecenal	0.397 ± 0.068 a	N.D. b	0.348 ± 0.013 a	N.D. b	N.D. b	N.D. b	6.04 × 10^−11^	4826-62-4
14.726	1400.00	Tetradecane	0.559 ± 0.002 abc	0.533 ± 0.007 bc	0.560 ± 0.021 ab	0.560 ± 0.002 abc	0.574 ± 0.240 a	0.564 ± 0.077 c	7.83 × 10^−2^	000629-59-4
14.909	1419.12	Tetradecanal	0.660 ± 0.005 a	0.359 ± 0.001 b	N.D. c	N.D. c	N.D. c	N.D. c	4.39 × 10^−10^	000124-25-4
16.383	1509.89	1-Hexadecanol	0.961 ± 0.004 a	N.D. c	0.935 ± 0.002 a	0.673 ± 0.001 b	N.D. c	N.D. c	8.43 × 10^−5^	36653-82-4
16.719	1540.38	4-methyl-Heptadecane	0.659 ± 0.036 a	N.D. b	N.D. b	N.D. b	N.D. b	N.D. b	3.28 × 10^−8^	26429-11-8
18.204	1600.00	Hexadecane	0.761 ± 0.042 b	0.760 ± 0.030 b	0.789 ± 0.033 b	0.788 ± 0.032 b	0.902 ± 0.199 a	0.968 ± 0.021 a	2.31 × 10^−5^	000544-76-3
19.123	1634.95	2-Pentadecanone	0.840 ± 0.100 a	N.D. b	N.D. b	N.D. b	N.D. b	N.D. b	1.34 × 10^−6^	2345-28-0
20.741	1642.73	Tetradecanoic acid	0.773 ± 0.001 a	0.272 ± 0.007 b	0.807 ± 0.057 a	0.757 ± 0.011 a	N.D. c	N.D. c	8.39 × 10^−9^	000544-63-8
21.334	1700.00	Heptadecane	1.229 ± 0.066 a	1.104 ± 0.515 ab	1.065 ± 0.057 bc	0.942 ± 0.198 c	1.043 ± 0.210 bc	1.153 ± 0.081 ab	7.26 × 10^−3^	000629-78-7
21.413	1900.00	Nonadecane	0.461 ± 0.014 a	N.D. b	N.D. b	N.D. b	N.D. b	N.D. b	1.01 × 10^−9^	629-92-5
22.268	1942.36	1-Octadecene	1.591 ± 0.046 a	1.263 ± 0.263 b	0.775 ± 0.148 c	0.035 ± 0.001 e	0.645 ± 0.102 d	N.D. e	1.97 × 10^−11^	112-88-9
22.865	1984.96	2-Nonadecanone	0.949 ± 0.002 bc	0.211 ± 0.27 d	1.015 ± 0.104 ab	1.042 ± 0.131 abc	0.913 ± 0.004 c	1.135 ± 0.090 a	6.70 × 10^−8^	000629-66-3
23.470	2026.34	n-Hexadecanoic acid	1.131 ± 0.159 a	1.085 ± 0.184 b	N.D. c	N.D. c	N.D. c	N.D. c	8.56 × 10^−8^	000112-62-9
23.811	2088.36	Hexadecenoic acid	1.067 ± 0.018 a	N.D. e	0.960 ± 0.006 ab	0.962 ± 0.007 b	0.748 ± 0.024 c	0.162 ± 0.069 d	1.11 × 10^−13^	000057-10-3
24.300	2144.53	Hexadecanoic acid, ethyl ester	N.D. c	N.D. c	1.043 ± 0.020 b	1.135 ± 0.002 a	N.D. c	N.D. c	3.36 × 10^−6^	000112-95-8
26.376	2177.57	Oleic acid	1.120 ± 0.013 ab	N.D. e	1.150 ± 0.053 a	1.099 ± 0.102 b	0.731 ± 0.008 c	0.439 ± 0.004 d	2.40 × 10^−9^	000060-33-3
28.870	2228.92	Octadecanoic acid	0.745 ± 0.001 d	N.D. e	1.599 ± 0.004 a	1.224 ± 0.010 b	0.855 ± 0.002 c	0.771 ± 0.095 d	1.10 × 10^−10^	9011-21-6
30.558	2262.71	Cyclotetracosane	1.279 ± 0.223 a	1.239 ± 0.089 a	1.068 ± 0.021 b	0.935 ± 0.002 c	0.545 ± 0.011 d	N.D. e	3.44 × 10^−11^	000297-03-0
31.879	2304.94	11-Tricosene	1.335 ± 0.071 a	1.110 ± 0.012 b	1.311 ± 0.081 a	1.304 ± 0.072 a	1.169 ± 0.002 b	0.895 ± 0.107 c	6.06 × 10^−5^	052078-56-5
33.506	2352.39	1-Docosene	1.300 ± 0.051 a	N.D. b	N.D. b	N.D. b	N.D. b	N.D. b	1.03 × 10^−9^	001599-67-3
34.741	2400.00	Tetracosane	1.539 ± 0.019 a	1.096 ± 0.023 c	1.410 ± 0.126 b	1.560 ± 0.128 a	0.690 ± 0.002 d	N.D. e	3.09 × 10^−11^	000646-31-1
36.627	2687.78	Squalene	1.236 ± 0.007 b	1.747 ± 0.008 a	1.710 ± 0.020 a	1.738 ± 0.091 a	1.749 ± 0.017 a	1.694 ± 0.012 a	5.37 × 10^−7^	007683-64-9
42.658	2924.03	Sitosterol	1.756 ± 0.036 a	N.D. c	0.629 ± 0.015 b	N.D. c	N.D. c	N.D. c	3.42 × 10^−11^	000083-46-5

^1^ RT: Retention time. ^2^ RI(*Exp*): Retention index determined by calculated using n-alkane standard C_7_-C_40_. ^3^ N.D.: Not detected. ^4^ SD: Standard deviation. Values represent the means of three replicates, and values within the same row with different letters are significantly different (*p* < 0.05).

**Table 3:** Pathway analysis results from the *B. dorsalis* (Diptera: Tephritidae) larvae metabolomics for the CON vs. IR, CON vs. HY, CON vs. Sup-HY, CON vs. HY + IR, and CON vs. Sup-HY + IR groups.

Group	Pathway	Hits	Expect	*p*-Value	Holm P	FDR
CON vs. IR	Biosynthesis of unsaturated fatty acids	3	0.117	0.000115	0.00963	0.00963
	Fatty acid biosynthesis	2	0.153	0.00864	0.717	0.363
	Fatty acid elongation	1	0.124	0.118	1	1
	Fatty acid degradation	1	0.127	0.121	1	1
	Steroid biosynthesis	1	0.137	0.13	1	1
CON vs. HY	Biosynthesis of unsaturated fatty acids	2	0.0938	0.00311	0.261	0.261
	Steroid biosynthesis	1	0.109	0.105	1	1
	Fatty acid biosynthesis	1	0.122	0.117	1	1
CON vs. Sup-HY	Biosynthesis of unsaturated fatty acids	1	0.0469	0.0463	1	1
	Fatty acid biosynthesis	1	0.0612	0.0603	1	1
CON vs. HY + IR	Biosynthesis of unsaturated fatty acids	3	0.141	0.000226	0.019	0.019
	Steroid biosynthesis	2	0.164	0.0102	0.848	0.356
	Fatty acid biosynthesis	2	0.184	0.0127	1	0.356
	Fatty acid elongation	1	0.148	0.14	1	1
	Fatty acid degradation	1	0.152	0.143	1	1
CON vs. Sup-HY + IR	Biosynthesis of unsaturated fatty acids	2	0.0703	0.00158	0.133	0.133
	Fatty acid elongation	1	0.0742	0.0724	1	1
	Fatty acid degradation	1	0.0762	0.0743	1	1
	Steroid biosynthesis	1	0.0820	0.0799	1	1
	Fatty acid biosynthesis	1	0.0918	0.0891	1	1

## Data Availability

All data are contained within the article.

## Supplementary Materials

The following supporting information can be downloaded at: https://www.mdpi.com/article/10.3390/insects15030177/s1, Figure S1: Metabolites identified in *B. dorsalis* (Diptera: Tephritidae) larvae exposed to different modified atmospheres under phytosanitary irradiation dose. The  represented compounds that were selected based on a significant *p*-value threshold (<0.05), while the  indicated non-significant compounds.
