# Supplementary material for: Development and Metabolomic Profiles of Bactrocera dorsalis (Diptera: Tephritidae) Larvae Exposed to Phytosanitary Irradiation Dose in Hypoxic Environment Using DI-SPME-GC/MS
Source: Insects. 2024 Mar 6;15(3):177. doi: 10.3390/insects15030177 (PMC10971714; doi:10.3390/insects15030177)
Supplement: Supplementary file 1 [file insects-15-00177-s001.zip › insects-2712437-supplementary.pdf]

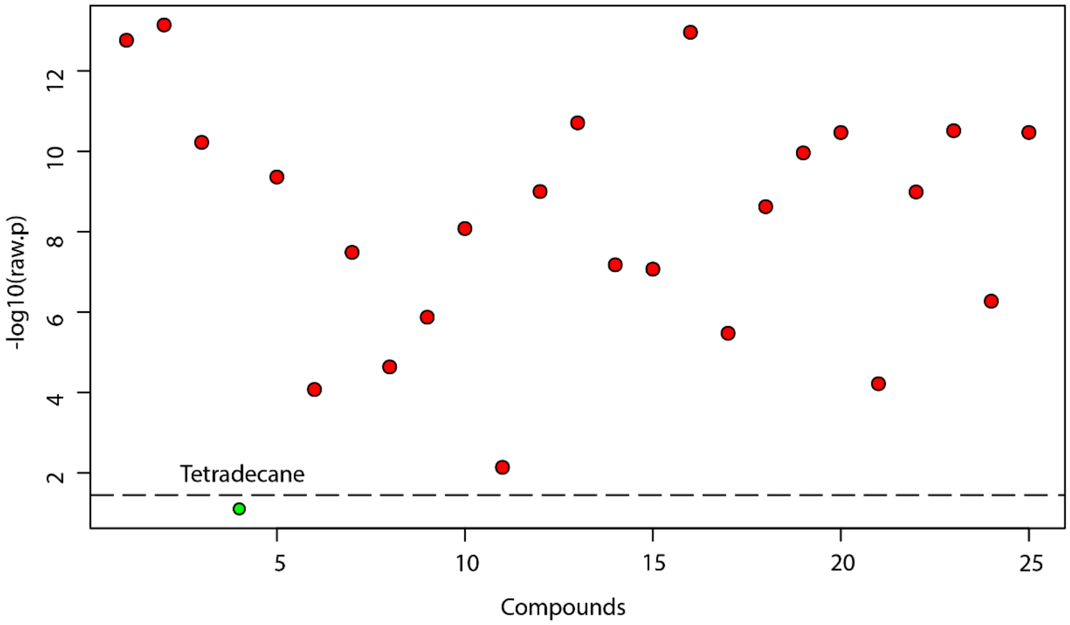

Figure S1. Metabolites identified in *B. dorsalis* (Diptera: Tephritidae) larvae exposed to different modified atmospheres under phytosanitary irradiation dose. The ● represented compounds that were selected based on a significant *P*−value threshold (< 0.05), while the ● indicated non−significant compounds.
